# Genome-Wide Identification and Expression Analysis of the bZIP Transcription Factors in the Mycoparasite *Coniothyrium minitans*

**DOI:** 10.3390/microorganisms8071045

**Published:** 2020-07-14

**Authors:** Yuping Xu, Yongchun Wang, Huizhang Zhao, Mingde Wu, Jing Zhang, Weidong Chen, Guoqing Li, Long Yang

**Affiliations:** 1 State Key Laboratory of Agricultural Microbiology and Hubei Key Laboratory of Plant Pathology, Huazhong Agricultural University, Wuhan 430070, China; yupingxu@webmail.hzau.edu.cn (Y.X.); wangyongchunhazu@163.com (Y.W.); huizhang1029@yeah.net (H.Z.); mingde@mail.hzau.edu.cn (M.W.); zhangjing1007@mail.hzau.edu.cn (J.Z.); guoqingli@mail.hzau.edu.cn (G.L.); 2 U.S. Department of Agriculture, Agricultural Research Service, Washington State University, Pullman, WA 99164, USA; w-chen@wsu.edu

**Keywords:** mycoparasitism, *Sclerotinia sclerotiorum*, intron arrangement, biological control

## Abstract

The basic leucine zipper (bZIP) proteins family is one of the largest and most diverse transcription factors, widely distributed in eukaryotes. However, no information is available regarding the *bZIP* gene family in *Coniothyrium minitans*, an important biocontrol agent of the plant pathogen *Sclerotinia sclerotiorum*. In this study, we identified 34 *bZIP* genes from the *C. minitans* genome, which were classified into 8 groups based on their phylogenetic relationships. Intron analysis showed that 28 *CmbZIP* genes harbored a variable number of introns, and 15 of them shared a feature that intron inserted into the bZIP domain. The intron position in bZIP domain was highly conserved, which was related to recognize the arginine (R) and could be treated as a genomic imprinting. Expression analysis of the *CmbZIP* genes in response to abiotic stresses indicated that they might play distinct roles in abiotic stress responses. Results showed that 22 *CmbZIP* genes were upregulated during the later stage of conidial development. Furthermore, transcriptome analysis indicated that *CmbZIP* genes are involved in different stages of mycoparasitism. Among deletion mutants of four *CmbZIP*s (*CmbZIP*07, -09, -13, and -16), only Δ*CmbZIP16* mutants significantly reduced its tolerance to the oxidative stress. The other mutants exhibited no significant effects on colony morphology, mycelial growth, conidiation, and mycoparasitism. Taken together, our results suggested that *CmbZIP* genes play important roles in the abiotic stress responses, conidial development, and mycoparasitism. These results provide comprehensive information of the *CmbZIP* gene family and lay the foundation for further research on the *bZIP* gene family regarding their biological functions and evolutionary history.

## 1. Introduction

*Coniothyrium minitans* Campbell is a mycoparasite of the phytopathogenic fungus *Sclerotinia sclerotiorum* [1,2]. It can parasitize hyphae and sclerotia of *S. sclerotiorum* and its related species, such as *S. trifoliorum* [3] and *S. minor* [4]. *C. minitans* has become a promising biocontrol agent due to its characteristics to obligate mycoparasite of *Sclerotinia* spp., long-term effects, and no pathogenicity to plants [3,4,5]. Now, *C. minitans* has been registered as a biocontrol agent for control of *Sclerotinia* diseases in many countries, such as Germany, Hungary, Russia, the United States [2,6].

Mycoparasitism is an important mechanism of *C. minitans* against *S. sclerotiorum* [4]. Fungal cell wall-degrading enzymes secreted by *C. minitans*, e.g., chitinase, β-1, 3-glucanase, and protease, have been proved to play important roles in mycoparasitism [7,8,9]. Both the yield and the activities of these cell wall-degrading enzymes were regulated by ambient pH [8]. Further studies have showed that *C. minitans* regulated pH by degradation of oxalic acid (OA) secreted by *S. sclerotiorum* [9]. Lou and colleagues [10] reported that *CmpacC*, a zinc finger transcription factor *pacC* homologue, positively regulated mycoparasitism and negatively regulated degradation of OA and antibiosis of *C. minitans.* However, our understanding of the regulatory networks involved in mycoparasitism of *C. minitans* is still incomplete.

Transcription factors play crucial roles in regulatory networks. They regulate target gene expression by binding to specific sites in the promoter regions. Basic leucine zipper (bZIP) protein is a category of key transcription factors unique to eukaryotes, and this gene family plays an important role in the process of development and various stress responses. Typical feature of bZIP transcription factor is the bZIP domain, which consists of 60–80 amino acids. The bZIP domain harbors two functionally distinct parts, a highly conserved basic region and a variable leucine-zipper region [11]. The basic region consists of about 16 amino acid residues with an invariant N-x7-R/K domain that mediates sequence-specific DNA binding and nuclear localization. The leucine-zipper region is composed of several repeats of leucine or other bulky hydrophobic amino acids and exactly arranged nine amino acid residues toward the C-terminus, that is necessary for homo- and/or heterodimerization of bZIP proteins [11,12,13,14].

The bZIP transcription factors are numerous and powerful in animals, plants, and microbes. There are 53 bZIP motifs in the *Homo sapiens* genome [15]. Activating transcription factor-2 (ATF-2) is a member of bZIP protein family in mammals, which plays a critical role in cell cycle proliferation and development, stress response, as well as response to DNA damage. Moreover, ATF-2 can tightly control the process of the oncogenic and tumor suppressor [16,17].

So far, the bZIP family members of various plants, such as 75 in Arabidopsis [18], 89 in rice [19], 92 in sorghum [20], 55 in grapevine [21], 114 in apple [22], 160 in soybean [23], have been predicted or identified. A large number of studies indicated that bZIP proteins of different subgroups are involved in multiple functions in plants, including abscisic acid sensitivity [24,25,26,27], regulating cell elongation [28], responding to drought, high salinity and low-temperature stresses [26,29,30,31,32], light signal transduction [33,34], sugar signaling [35,36,37], and resistance to pathogen infection [38,39].

Meanwhile, many critical bZIP proteins have been identified in fungi. For example, YAP1 is required for oxidative stress tolerance [40,41,42,43], Atf1 affected osmotic stress [44,45,46,47], FlbB mediated asexual development and gliotoxin production [48,49], Hac1 answered for unfolded protein response [50,51,52,53] MeaB involved in nitrogen metabolite repression [52,53,54,55,56], and so on. These results indicated that the bZIP transcription factors have diverse functions and conservative evolution.

At present, the bZIP family members of few model fungi were clarified on a genome-wide scale, such as *Saccharomyces cerevisiae* [56], *Magnaporthe oryzae* [52,53], and *Ustilaginoidea virens* [57]. However, the genome-wide analysis of the bZIP gene family in biocontrol fungi has not been reported.

In this study, we report the identification and characterization of 34 bZIP genes from *C. minitans*. We analyzed expression of the CmbZIP genes in different stages of conidial development and mycoparasitism, as well as in response to abiotic stresses. To elucidate the functions of CmbZIPs, knockout mutants of four CmbZIPs (CmbZIP07, -09, -13, and -16) were generated by the split-marker strategy. Functional analysis suggested that only CmbZIP16 played an important role in oxidative stress tolerance. These results provide foundation for further investigation into the biological functions of the bZIP transcription factor genes in *C. minitans*. 

## 2. Materials and Methods 

### 2.1. Fungal Strains and Cultural Media

Two fungal strains, *C. minitans* Chy-1 and *S. sclerotiorum* A5, were used in this study. The strains Chy-1 and A5 were described in the previous study [8]. PDA (potato dextrose agar, 200 g potato, 20 g dextrose, 24 g agar, and 1 L water) and PDB (potato dextrose broth, 200 g potato, 20 g dextrose, and 1 L water) were used for culturing *C. minitans* and *S. sclerotiorum*. Both fungi were incubated at 20 °C in the dark. To prepare the conidial suspensions, conidia of *C. minitans* Chy-1 were harvested from 14-day-old colonies with sterile distilled water and the conidial concentration in the suspensions was determined using a hemacytometer.

### 2.2. Identification of the bZIP Transcription Factors in C. minitans

To identify all *bZIP* genes in *C. minitans*, local BLASTp was carried out. Genome of *C. minitans* downloaded from the NCBI database (the GenBank accession code VFEO00000000) [58] was set as a database; bZIP domain of 110 registered bZIP proteins from *M. oryzae* (with 22 registered bZIP proteins), *S. cerevisiae* (with 17 registered bZIP proteins), and the expanded bZIP transcription factor family in *Phytophthora sojae* (with 71 registered bZIP proteins) were used as query with an e-value cutoff 1e^−5^ [52,59]. All output genes were further confirmed in the online software CDD (https://www.ncbi.nlm.nih.gov/Structure/cdd/wrpsb.cgi) and SMART (http://smart.embl-heidelberg.de/smart/set_mode.cgi?NORMAL=1). The final selected candidate genes were confirmed as positive by at least one of these two tools.

### 2.3. Multiple Sequence Alignment and Phylogenetic Analysis

Amino acid sequences of the 34 identified bZIP domains were aligned with those from other representative fungi using software MUSCLE V3.8.1551 with slight modifications. The amino acid sequences of 125 *bZIP* genes from seven representative fungi (17 from *Aspergillus nidulans*, 22 from *M. oryzae*, 17 from *Neurospora crassa*, 17 from *S. sclerotiorum*, 9 from *Ustilago maydis*, 15 from *S. cerevisiae*, and 28 from *U. virens*) were download from NCBI (https://www.ncbi.nlm.nih.gov/) or FTFD (http://ftfd.snu.ac.kr/index.php?a=view). A phylogenetic tree was constructed using IQ-TREE Multicore version 1.6.12 with the maximum-likelihood method, and the bootstrap test was carried out with 1000 iterations.

### 2.4. Sequence Analysis and Gene Structural Characterization

The number of amino acids, molecular weights, and theoretical isoelectric points (pI) of the selected candidates were analyzed using the ExPASy proteomics server (http://web.expasy.org/protparam/). Both genomic sequence of *bZIP* genes and the corresponding CDS sequence were submitted to the Gene Structure Display Server (GSDS version 2.0, http://gsds.cbi.pku.edu.cn/) to show the number and arrangement of intron and exon.

Additionally, the online software, Multiple Em (Expectation Maximization) for the Motif Elicitation tool (MEME version 5.1.0, http://alternate.meme-suite.org/tools/meme) was used to recognize additional conserved motifs apart from the bZIP domain of the bZIP transcription factors in *C. minitans*. The parameters of the motif discovery mode, site distribution, number of motifs, minimum width, maximum width, were set as classic mode, any number of repetitions, 20, 6, and 200, respectively.

### 2.5. Culture Conditions, Biological Samples Collection, and Total RNA Extractions

For abiotic stress treatments, 3-day-old mycelia of *C. minitans* in PDB were collected, and then treated with different abiotic stress factors for 4 h. Nine abiotic stress factors were tested, including ionic stress (NaCl, 1 mol L^−1^), osmotic stress (sorbitol, 1 mol L^−1^), cell wall stress (SDS, 0.01%), two kinds of redox stress (H_2_O_2_, 10 mmol L^−1^; DTT, 1.5 mmol L^−1^), two levels of pH stress (pH 3, adjusted by 4 mmol L^−1^ HCl and pH 10, adjusted by 4 mmol L^−1^ NaOH), and two levels of temperature stress (low temperature, 4 °C and high temperature, 37 °C). Mycelia without any abiotic stress treatment (named PDB) was designated as the control. At the end of stress treatments, mycelia were collected by filtration through sterilized gauze and used for RNA extractions. Each treatment contained three replicates, and the entire experiment was repeated three times.

To investigate the expression patterns of the *bZIP* genes in different stages of conidial development, aliquots (100 μL) of the conidial suspension of *C. minitans* (1 × 10^7^ conidia mL^−1^) were inoculated on autoclaved cellophane films placed on PDA plates. After the incubation at 20 °C for 48, 60, 72, 84, and 96 h, mycelia were harvested for RNA extractions. Each treatment contained three replicates, and the entire experiment was repeated three times.

Total RNA was extracted from the mycelial samples using E.Z.N.A. Fungal RNA Kit (TaKaRa Co., Dalian, China), following the manufacturer’s instructions. Agarose gel electrophoresis and Nano Drop 1000 Spectrophotometer (Thermo Scientific, Waltham, MA, USA) was used to check the quality and estimate concentration of the total RNA.

### 2.6. Reverse Transcription and Fluorescence Quantitative Polymerase Chain Reaction (RT-qPCR)

The cDNA was synthesized using a PrimeScript™ RT reagent Kit with gDNA Eraser (TaKaRa Co., Dalian, China). RT-qPCR was performed by using TB Green™ Premix Ex Taq™ II (TaKaRa Co., Dalian, China), and the actin gene *Cmactin* was used as reference gene in each PCR assay [9]. All samples were amplified in triplicate and each experiment was performed three times independently. All primers used in this experiment are listed in Table S1. The relative expression was calculated by using ΔΔCt method [ΔΔCt = (Ct_target gene_ − Ct_Actin gene_)_treatment_ − (Ct_target gene_ − Ct_Actin gene_)_control_]. We used the absolute value of log_2_Ratio (treatment/control) > one fold change to judge the significance of gene expression differences. TBtools (v0.66836) served to display the relative expression in heatmap.

### 2.7. RNA-Sequencing (RNA-seq) Data Analysis

In order to analyze the expression patterns of the *CmbZIP* genes during the mycoparasitism period, RNA-sequencing was conducted. Aliquots (100 μL) of the conidial suspension of *C. minitans* (1 × 10^8^ conidia mL^−1^) were mixed with 100 μL 2-day-old hyphae fragments of *S. sclerotiorum*, and then inoculated on sterile cellophane films placed on WA (water agar) plates. After the incubation at 20 °C for 0, 1, 3, 6, and 8 d, mycelia were harvested for total RNA extractions. There were three replicates for each treatment. A total of 15 RNA samples were extracted separately for sequencing on Illumina HiSeq X Ten platform in Biomarker Technologies Corporation (Beijing, China). For cluster displays, the data convenient, original FPKM (Fragments Per Kilobase Million) values were divided by the mean of all of the values, and the ratios were transformed by log2. The absolute value of log_2_Ratio (treatment/control) > 1.0 was regarded as the threshold to judge the significance of gene expression differences. The heatmap was made by TBtools (v0.66836).

### 2.8. Disruption of Four CmbZIP Genes

Four *CmbZIP* genes (*CmbZIP07*, *-09*, *-13,* and *-16*) were disrupted using the split marker system [60]. The schematic diagram of the deletion strategy is outlined in Figure S1A. The 5′ and 3′ flanking sequences of each *CmbZIP* gene were amplified with the primers listed in Table S1 and then fused with part of the hygromycin fragment. Two split-maker DNA fragments for each gene were transformed into protoplasts of the wild type of *C. minitans,* respectively, using the PEG-mediated transformation technique [9]. The deletion transformants were screened on PDA plates containing hygromycin B (50 µg mL^−1^) for three times and verified by PCR using four primer sets (Veri-*bZIPx*-5′-F and Veri-5′-R, *HYG*-F and *HYG*-R, Veri-3′-F and Veri-*bZIPx*-3′-R, *CmbZIPx*-F and *CmbZIPx*-R) (Figure S1A). Veri-*bZIPx*-5′-F and Veri-5′-R and Veri-3′-F and Veri-*bZIPx*-3′-R were used to test whether the 5′ flanking sequences and 3′ flanking sequences of *CmbZIP* genes had homologous recombined with hygromycin phosphotransferase gene (*HYG*) or not, respectively. *HYG*-F and *HYG*-R were used for detecting whether the two fragments of the overlapping marker gene (*HYG*) had homologous recombined with each other or not. *CmbZIPx*-F and *CmbZIPx*-R were used for checking whether the target gene had been replaced by the overlapping marker gene (*HYG*) or not.

### 2.9. Phenotypic Characterization

The *bZIP* gene disruption mutants were characterized for colony morphology, growth rate, conidial production, and parasitic ability as described before [61]. The sensitivities of the mutants to different abiotic stresses were examined as described by Lou et al. [10]. The abiotic stress agents tested include 5 mmol L^−1^ H_2_O_2_, 0.1 mmol L^−1^ VK3 (vitamin K3), 16 mmol L^−1^ OA, 1.5 mmol L^−1^ DTT (dithiothreitol), and 200 μg mL^−1^ CFW (calcofluor white).

## 3. Results

### 3.1. Identification of bZIP Genes in C. minitans 

In order to identify *bZIP* genes in the *C. minitans*, we used local BLASTp to search the *C. minitans* genome database using previously published bZIP protein domain sequences as queries. Thirty-four nonredundant sequences were identified using an E-value < 0.1 from database and confirmed using the CDD and SMART software. The 34 *C. minitans bZIP* genes were named from *CmbZIP01* to *CmbZIP34.* The lengths of the 34 CmbZIP proteins ranged from 123 (CmbZIP21) to 1580 (CmbZIP04) amino acids, and the theoretical molecular weights varied from 13.5 (CmbZIP21) to 175.3 (CmbZIP04) kDa, with predicted pI values in the range of 4.85–10.00 (Table S2).

### 3.2. Multiple Sequence Alignment and Phylogenetic Analysis of bZIP Gene Family

**Figure 1:**
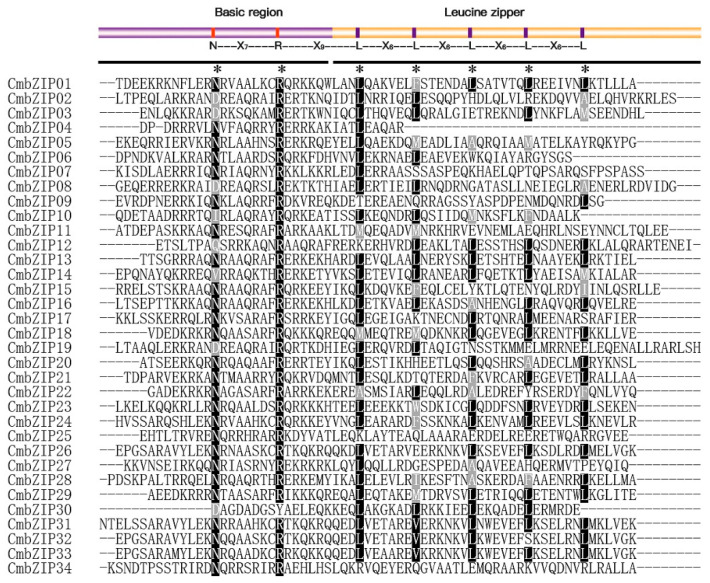
Multiple sequence alignment of CmbZIP domains. Amino acid sequences of CmbZIP domains are aligned by MUSCLE V3.8.1551 with minor modifications. The model and invariant of the basic region and leucine zipper are shown in the upper part. Asterisks show the conserved amino acids of bZIP domain. White letters in black shadow indicate conserved amino acid residues in right position, and gray shadow is for the position replaced by other less conserved amino acids.

The conventional bZIP domain harbors a highly conserved basic region with an invariant N-X_7_-R/K motif and a variable leucine-zipper region or several heptad repeats of other bulky hydrophobic amino acids [31]. To clarify the characteristics of the predicted bZIP domains in *C. minitans*, amino acid sequences were aligned with the typical bZIP domains. As illustrated in Figure 1, most of the candidate bZIP domains shared the conventional pattern of invariant residues (N-X_7_-R/K) and the leucine-zipper region. Exceptions were detected in four *CmbZIP* genes. Three CmbZIPs (CmbZIP09, -25, and -34) contained the basic region, but the leucine zipper region lacked the heptad repeats of the leucine (L) or other bulky hydrophobic amino acids. On the other hand, CmbZIP30 had the complete leucine zipper region, but the basic region lacked the invariant N-X7- R/K motif. The invariant N-X_7_-R motif was more popular than the N-X_7_-K motif, indicating that arginine (R) was more conserved than Lysine (K) in the CmbZIP domains. Unexpectedly, the core asparagine (N) residue in 8 out of 34 (23.5%) CmbZIP domains were replaced by aspartic acid (D), isoleucine (I), glutamine (Q), or valine (V). For the part of leucine zipper, the first leucine (L) was more conserved than the following leucine (L), which had been frequently substituted with other bulky hydrophobic amino acids, such as methionine (M), alanine (A), and isoleucine (I).

**Figure 2:**
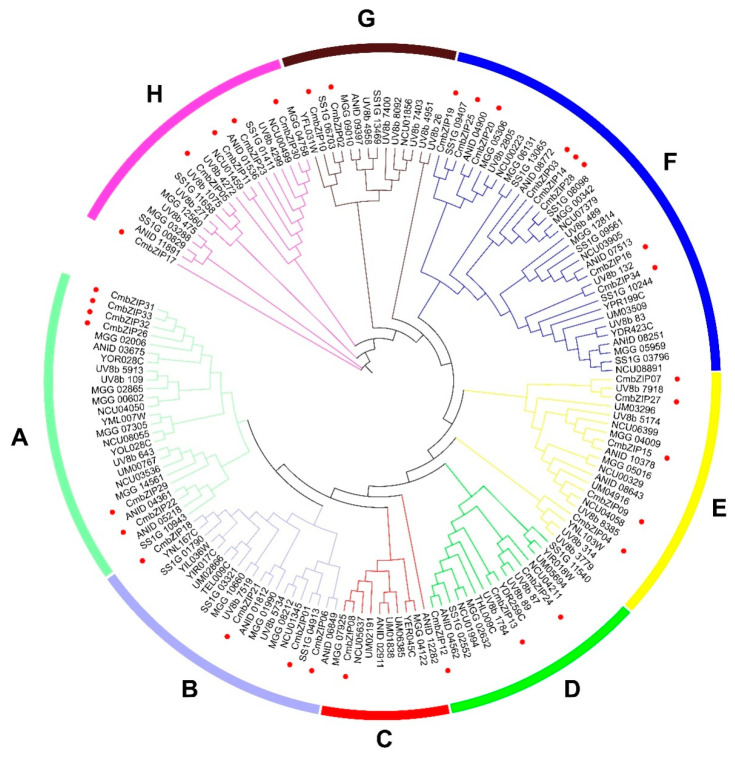
Phylogenetic analysis of bZIP proteins from eight fungi. The maximum-likelihood trees were constructed based on 159 full-length protein sequences. The phylogenetic tree was constructed using IQ-TREE multicore version 1.6.12 and the bootstrap test carried out with 1000 iterations. The proteins are clustered into eight clades (A–H) indicated by colored branches. The CmbZIPs are denoted by red circles. The sequences were collected from organisms as follows: Cm, *Coniothyrium minitans*; MGG, *Magnaporthe oryzae*; ANID, *Aspergillus nidulans*; UV8b, *Ustilaginoidea virens*; NCU, *Neurospora crassa*; UM; *Ustilago maydis*; and SS1G; *Sclerotinia sclerotiorum*; the rest proteins were from *Saccharomyces cerevisiae*.

To investigate the evolutionary relationship among the *CmbZIP* genes and other fungal *bZIP* genes, a phylogenetic analysis was constructed based on a total 159 bZIP proteins including 34 CmbZIP proteins and 125 selected other fungal bZIP proteins (Figure 2). The 159 *bZIP* genes were divided into eight clades (designated as A to H). In general, the *bZIP* genes from 8 fungi including the 34 *CmbZIP* genes were distributed evenly to all these clades, indicating that these *bZIP* genes were evolved before divergence of these fungi. Four *CmbZIP* genes (*CmbZIP26*, *CmbZIP31*, *CmbZIP32,* and *CmbZIP33*) in clade A were closer than other bZIPs, suggesting that a genome duplication event had been happened in recent period.

### 3.3. Intron Distribution Patterns and Insertion Sites in CmbZIP Genes 

**Figure 3:**
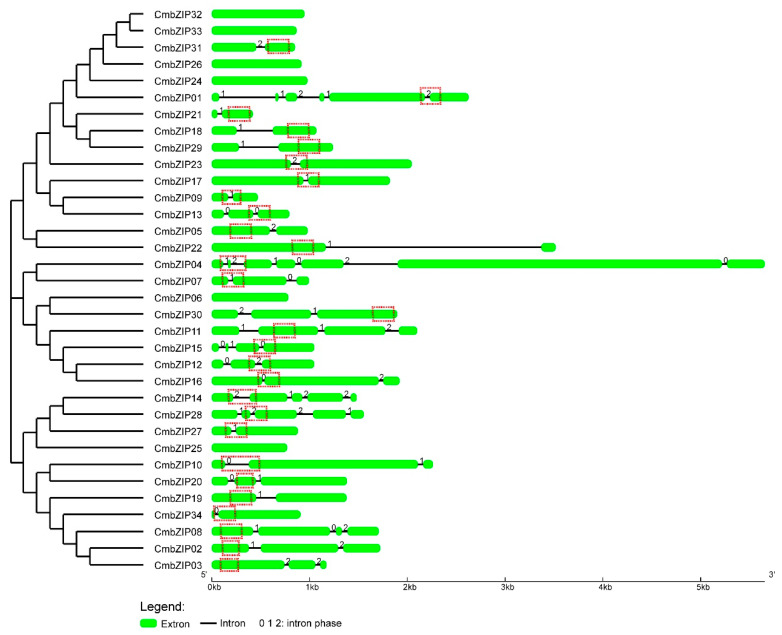
Analysis of gene structures of CmbZIPs. Arrangements of introns and exons are displayed by GSDS version 2.0. Red dotted boxes, representing the bZIP domains of each sequence, are marked manually. Green boxes indicate exons, drawn to scale. Black lines between exons represent introns. The number 0, 1, and 2 represent the splicing phase of intron. Phase 0 means intron splicing site is between codons, and phase 1 and phase 2 means the intron splicing site is located after the first and second nucleotide of a codon, respectively.

In order to illuminate the structural characteristics of *CmbZIP* genes, intron distribution patterns and insertion sites were determined using GSDS. Gene structure analysis showed that there are 28 *CmbZIP* genes with introns and 6 *CmbZIP* genes without intron (Figure 3). The number of introns in the 28 *CmbZIP* genes ranged from 1 to 6. Among them, 12 *CmbZIP* genes harbored 1 intron, 9 genes had 2 introns, 3 genes possessed 3 introns, 2 genes had 4 introns, and *CmbZIP01* and *CmbZIP04* had 5 and 6 introns, respectively. The length of introns ranged from 45 to 2207 base pairs (bp), and more than 68% of the introns were between 45 and 80 bp. All introns belonged to canonical GT-AG introns, except the fifth intron in *CmbZIP04* and the fourth intron in *CmbZIP28*. 

**Figure 4:**
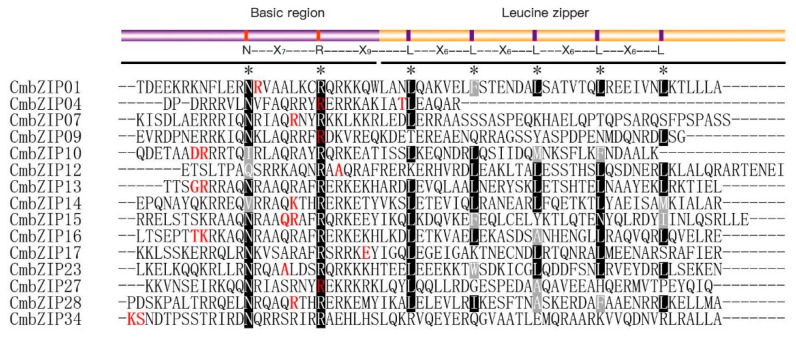
Analysis of splicing phase of intron in the bZIP domain of the CmbZIP proteins. Sequences handling is described as Figure 1. Splicing sites locate on the red letters. Single letter means splicing sites is located after the first or second nucleotide of a codon, and double letter means splicing sites is between two codons.

There is no preference for intron phase in the *CmbZIP* genes. However, more than half (15, 53.6%) of the *CmbZIP* genes with introns shared a feature that intron inserted into the bZIP domain (Figure 3). These 15 bZIP domains were with one intron, except *CmbZIP04* with two introns. In addition, the most intron insertion sites were located in the N-X_7_-R motif coding regions (Figure 4). Interestingly, 6 out of the 15 of the intron insertion sites were arginine (R), including 3 of the intron-insertion sites in the core arginine (R) in the N-X_7_-R motif. 

### 3.4. Verification of Additional Structural Features in the C. minitans bZIP Genes 

**Figure 5:**
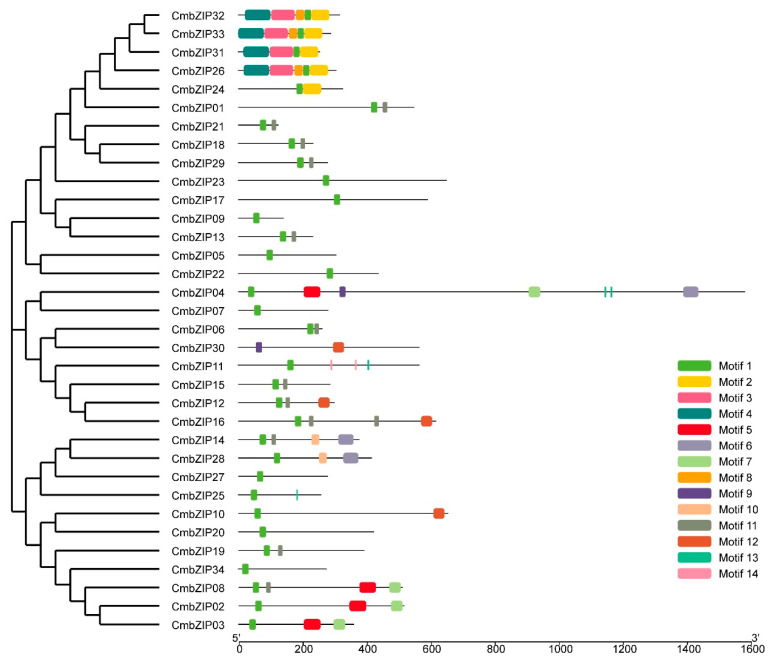
Distributions of conserved motifs in *CmbZIP* genes. MEME was used to recognize additional conserved motifs of bZIP transcription factors in *C. minitans*. Fourteen motifs are highlighted in different colored boxes. Multilevel consensus amino acid sequences of predicted conserved motifs are listed in Figure S2.

Previous study suggested that additional domains in the bZIP proteins endowed *bZIP* genes with different functions [62]. To gain insights into the additional structural features in the *CmbZIP* genes, sequences were analyzed by MEME. In total, 14 additional structural features, including the basic region, were identified in the CmbZIP proteins (Figure 5, Figure S2). The length of these conserved motifs ranged from 6 to 80 amino acids. Based on the multilevel consensus amino acid sequences of motifs, motif 1 represented the basic region and the first leucine (L) of leucine zipper. Motif 1 existed in all the CmbZIP proteins, except the CmbZIP30, consistent with the result of multiple sequence alignment. The consensus amino acid sequence of motif 11 was similar to the leucine zipper, which kept in step with motif 1 in many CmbZIP proteins. Motif 5 equaled to Pfam DUF3425 existed in CmbZIP02, CmbZIP03, CmbZIP04, and CmbZIP08. Four CmbZIP proteins (CmbZIP26, -31, -32, and -33) shared almost the same additional motifs (motif 1, motif 2, motif 3, motif 4, and motif 8), except CmbZIP31 without motif 8. This is consistent with the result of the phylogenetic analysis.

### 3.5. Expression Patterns of bZIP Genes Response to Abiotic Stress 

**Figure 6:**
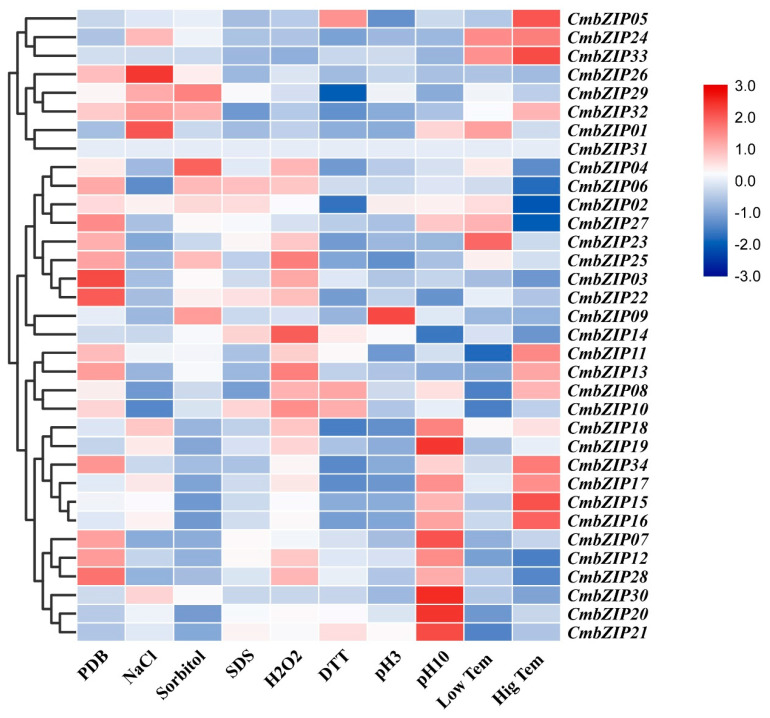
Expression profiles of *CmbZIP* genes response to abiotic stress. Relative expression values of RT-qPCR results were transformed by log2. Fold changes in gene expression are shown in color according to the scale. PDB: potato dextrose broth, NaCl: sodium chloride, SDS: sodium dodecyl sulfate, H_2_O_2_: hydrogen peroxide, DTT: dithiothreitol, Low Tem: low temperature (4 °C), Hig Tem: high temperature (37 °C).

To investigate the response of the *CmbZIPs* to various environmental stresses at transcriptional levels, the transcription patterns of *CmbZIPs* under different treatments were measured by RT-qPCR (Figure 6). Under NaCl treatment (1 mol L^−1^), three *CmbZIPs* (*CmbZIP01*, *-24*, and *-30*) were upregulated after 4 h treatment, whereas 13 *CmbZIPs* (*CmbZIP*0*3*, *-04*, *-06*, *-07*, *-08*, *-09*, *-10*, *-13*, *-22*, *-23*, *-25*, *-27*, and *-28*) were down-regulated. Under osmotic treatment (1 mol L^−1^ sorbitol), two *CmbZIPs* (*CmbZIP09* and *-24*) showed induction, while 8 *CmbZIPs* (*CmbZIP03*, *-07*, *-12*, *-13, -15, -23*, *-28*, and *-34*) were repressed. Under the cell wall stress treatment (0.01% SDS), there were no upregulated *CmbZIP* genes, but six were downregulated *CmbZIPs* (*CmbZIP03*, *-08*, *-13*, *-25*, *-26*, and *-32*). Under oxidative stress treatment (10 mmol L^−1^ H_2_O_2_), only *CmbZIP14* was induced and two *CmbZIPs* (*CmbZIP07* and *-32*) were repressed. On the contrary, only *CmbZIP05* was upregulated and 15 *CmbZIPs* (*CmbZIP02*, *-03*, *-04*, *-07*, *-09*, *-13*, *-22*, *-23*, -24, *-25*, *-26*, *-27*, *-29*, *-32*, and *-34*) were downregulated by reductive stress (1.5 mmol L^−1^ DTT). Under acid stress treatment (pH 3), only *CmbZIP09* showed induction, but 12 *CmbZIPs* (*CmbZIP03*, *-07*, -*11*, -*13*, *-22*, *-23, -25*, *-27, -28*, *-30, -32*, and *-34*) were repressed. On the other hand, four *CmbZIPs* (*CmbZIP01*, *-20*, *-21*, and *-30*) were upregulated and eight *CmbZIPs* (*CmbZIP03*, -*13*, *-14*, *-22, -23*, *-25, -26*, and *-32*) were downregulated under pH 10 treatment. Under cold treatment (4 °C), *CmbZIP01,* -*24*, and *-33* transcripts increased, and 11 *CmbZIPs* (*CmbZIP03*, -0*7*, *-08*, *-09, -10*, *-11, -12*, *-13*, *-22, -26*, and *-28*) transcripts decreased. On the other hand, 5 *CmbZIPs* (*CmbZIP05*, *-15*, *-16*, *-24*, and *-33*) were upregulated and 14 *CmbZIPs* (*CmbZIP02*, -0*3*, *-04*, *-06, -07*, *-09, -12*, -*14*, -*22*, -*23*, -*26*, -*27*, -*28*, and *-30*) were downregulated under heat treatment (37 °C).

### 3.6. Expression Patterns of bZIP Genes in Different Stages of Conidial Development 

**Figure 7:**
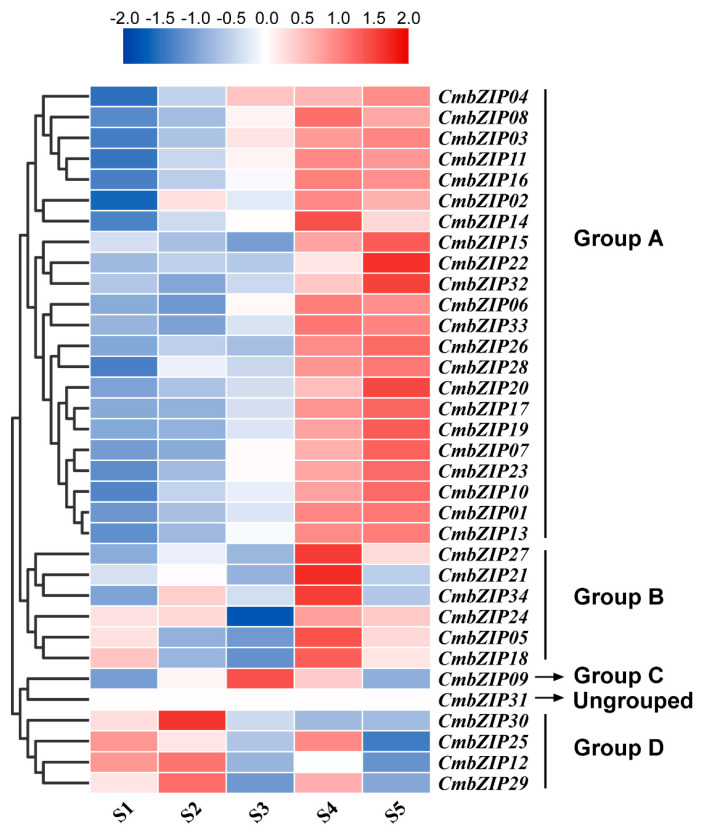
Expression profiles of *CmbZIP* genes under different stages of conidial development. S1: hyphal growth stage (48 hpi), S2: primordial formation stage (60 hpi), S3: pycnidial initiation stage (72 hpi), S4: pycnidial formation stage (84 hpi), S5: pycnidial maturation stage (96 hpi). Relative expression values of RT-qPCR results were transformed by log2. Blue and red boxes indicate lower and higher expression levels, respectively.

Previous studies have demonstrated that conidiogenesis is a critical step in the life cycle of *C. minitans*, and it has been divided into five stages, S1: hyphal growth stage (48 hpi), S2: primordial formation stage (60 hpi), S3: pycnidial initiation stage (72 hpi), S4: pycnidial formation stage (84 hpi), and S5: pycnidial maturation stage (96 hpi) [63,64]. To gain insight into the potential functions of the 34 *CmbZIP* genes in conidiogenesis, the expression pattern of *CmbZIP* genes during the five stages of conidial development were examined by RT-qPCR. Based on the gene expression profiles, except *CmbZIP31* whose expression was silent, all the *CmbZIP* genes could be divided into four groups (Figure 7). The group A including 22 genes (64.7%) was upregulated in the later stages (S4–S5) of conidial development, representing the major expression patterns of *CmbZIPs.* The group B included six genes, whose expression pattern was significantly induced in the S4 stage and decreased in the S5 stage. The group C included only the *CmbZIP09* gene, which was upregulated in the S3 stage and then downregulated at later stages. Finally, the group D included five genes, which were upregulated in the early stage (S1–S2) and downregulated in the later stages (S3–S5). These results indicated that individual *CmbZIP* genes were responsible for different stages of conidial development.

### 3.7. Transcriptome Analyses of bZIP Genes in the Process of Mycoparasitism

**Figure 8:**
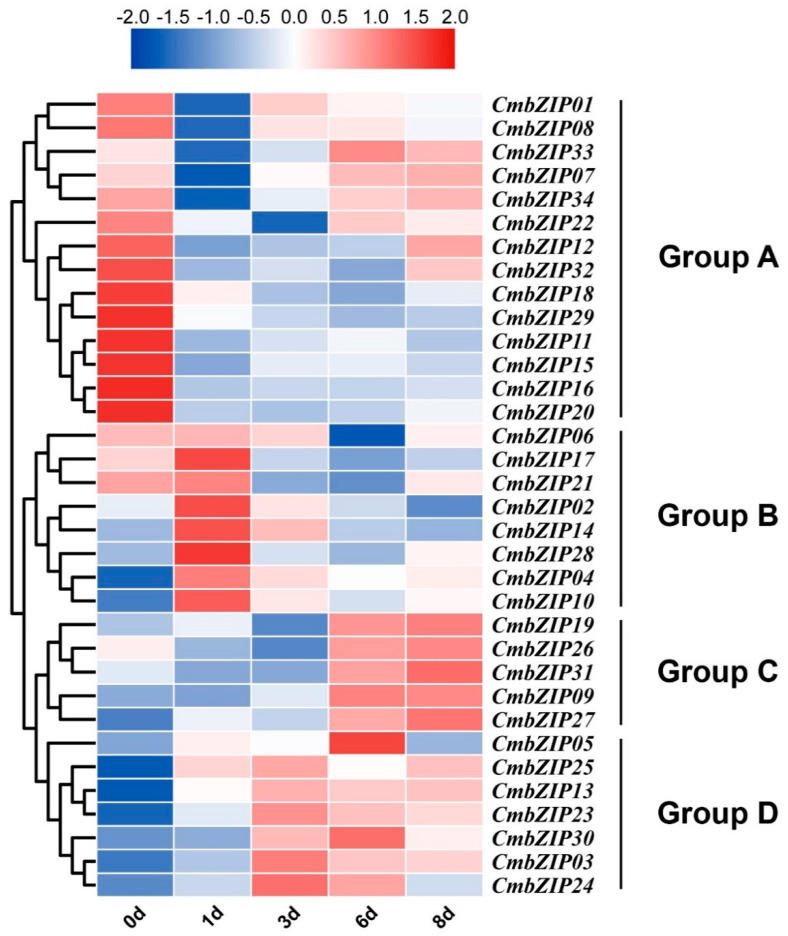
Expression profiles of *CmbZIP* genes in the process of mycoparasitism. Expression of *CmbZIP*s in the process of mycoparasitism as determined by RNA-Seq. The original FPKM values of *CmbZIP* genes were transformed by log2. The color scale represents the fold change in the gene expression value, i.e., red for upregulated and blue for downregulated.

To investigate the transcriptional pattern of the *CmbZIP* genes during the process of mycoparasitism, an RNA-seq approach was applied to analyze the expression of the *CmbZIP* genes at 1, 3, 6, and 8 days postinoculation. According to the gene expression profiles, four gene expression groups were obtained (Figure 8). The group A included 14 genes, all of which had high expression before inoculation, and then which were downregulated at 1 day postinoculation. On the contrary, the group B including six genes was upregulated at 1 day postinoculation. The group C contained five genes, all of which had high expression at 6 and 8 days postinoculation. The expression of seven genes in the group D was higher on the 3 and 6 days postinoculation. These results indicated that all the 34 *CmbZIP* genes are involved in different stages of mycoparasitism.

### 3.8. Function Analysis of Individual CmbZIP Genes

In order to characterize the functions of individual *CmbZIP* genes, we carried out gene knockout and analyzed the phenotypes of transformants. Since *CmbZIP09* was upregulated under acid stress treatment (pH3), *CmbZIP07*, -*13*, and -*16*, were significantly induced in the later stages (S4–S5) of conidial development and *CmbZIP09* and *-13* were upregulated in the later stages of the process of mycoparasitism, so these four *CmbZIP* genes (*CmbZIP07*, *-09*, *-13,* and *-16*) were selected for gene knockout assay. More than 100 transformants of each gene could subculture on PDA plates containing hygromycin B (50 µg mL^−1^) for three times, and at least two independent knockout mutants were obtained for each gene as confirmed by PCR results (Figure S1B).

**Figure 9:**
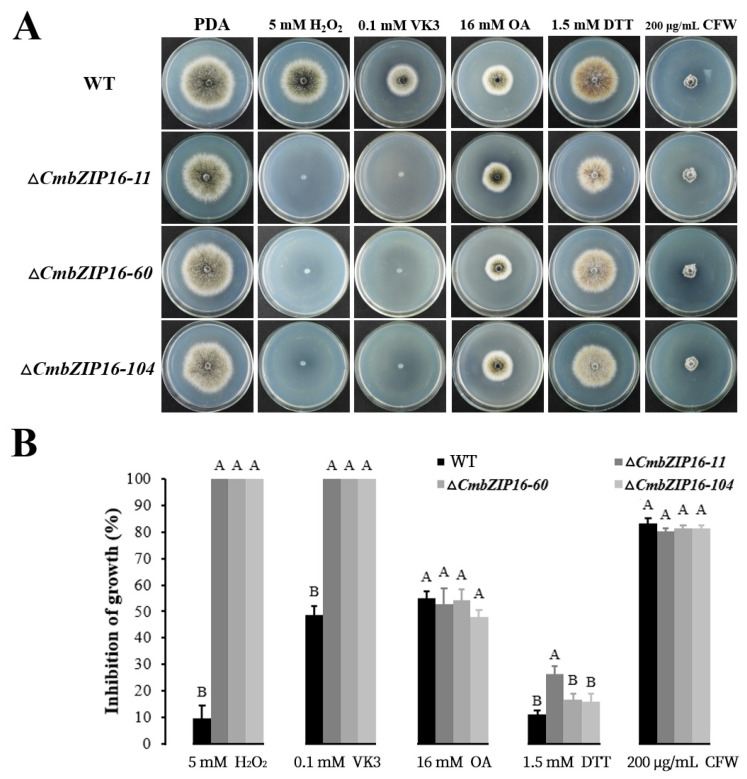
Sensitivity of Δ*CmbZIP16* mutants to abiotic stresses. (**A**) Morphology of wild type and Δ*CmbZIP16* mutants on PDA media with abiotic stresses (5 mmol L^−1^ H_2_O_2_, 0.1 mmol L^−1^ VK3, 16 mmol L^−1^ OA, 1.5 mmol L^−1^ DTT, and 200 μg mL^−1^ CFW), at 10 days after inoculation. (**B**) Inhibition of growth on PDA was examined under several separate stress factors. Values are means of three independent replicates and bars represent the standard deviation. Statistical analyses were performed using the one-way ANOVA method at the 99% significance level. PDA, potato dextrose agar; H_2_O_2_, hydrogen peroxide; VK3, vitamin K3; OA, oxalic acid; DTT, dithiothreitol; CFW, calcofluor white.

All of the four *CmbZIPs* mutants were examined for phenotypic changes in colony morphology, growth rate, conidial production, parasitic ability, and tolerance against the abiotic stress agents. Results showed that individual disruption of four *CmbZIP* genes had no significant effects on colony morphology, mycelial growth, conidiation, and mycoparasitism (data not shown). The Δ*CmbZIP16* mutants (Δ*CmbZIP16-11, -60, and -104*) showed hypersensitivity to the oxidative stress (5 mmol L^−1^ H_2_O_2_ and 0.1 mmol L^−1^ VK3) (Figure 9). However, the knockout mutants of other three *CmbZIP* genes (*CmbZIP-7*, *-9*, and *-13*) exhibited no appreciable difference with respect to the WT in response to the abiotic stresses, including 5 mmol L^−1^ H_2_O_2_, 0.1 mmol L^−1^ VK3, 16 mmol L^−1^ OA, 1.5 mmol L^−1^ DTT, and 200 μg mL^−1^ CFW (data not shown). 

## 4. Discussion

*C. minitans* is a promising biological control agent of the polyphagous phytopathogenic fungus *S. sclerotiorum*. Thus, by exploring the detail of the evolutionary history, the biological characteristics and the biocontrol mechanism of *C. minitans* could improve its effectiveness. With the rapid development of high throughput sequencing technology and availability of the genome sequence, studying gene families becomes more feasible [19,20,52,57]. Transcription factors (TFs) regulate downstream genes and enable a variety of critical cellular functions [10,43]. The bZIP TF family is one of the largest, most diverse, most ancient, and best characterized TF families, widely distributed in eukaryotes, with certain and different functions [15,18,23,53,59]. The *bZIP* gene family has been identified and characterized in a dozen of plant species [19,20,21,22,65], while little is known about bZIPs gene family in fungi, although a large number of individual *bZIP* genes with a specific function were identified [43,46,49,53]. So far, either individual *bZIP* genes or the *bZIP* gene family in *C. minitans* remains unknown. Consequently, it is a significant step to identify *bZIP* gene family in *C. minitans* also in order to promote the knowledge and enable future investigations for this fungal species.

In plants, previous studies showed that there were 75, 89, 92, 114, and 160 bZIP genes in *Arabidopsis*, rice, sorghum, apple, and soybean, respectively [19,20,22,23,33]. Otherwise, in fungi, there were 22 *bZIP* genes in *M. oryzae* [52], 28 *bZIP* genes in *U. virens* [57], and 34 *bZIP* genes in *C. minitans*. These data suggest that the number of *bZIP* genes in fungi is significantly less than those in plants, probably because the genome of fungi is smaller than that of plants. 

It has been reported that bZIP superfamily has evolved from a single ancestral eukaryotic gene and undergone multiple independent expansions [66]. In the phylogenetic tree performed in this study, 159 bZIP sequences distributed evenly to all 8 clades (A–H), indicating that the *bZIP* gene family existed prior to the divergence of these fungi. There must be a gene duplication event that had been happened in recent period in *C. minitans* because four *CmbZIP* genes (*CmbZIP26*, *-31*, *-32,* and *-33*) in the clade A were much closer than other *bZIP* genes and additionally, the gene structure of these four *CmbZIP* genes is very similar. They contained the same motifs (motif 1, motif 2, motif 3, motif 4, and motif 8), except for *CmbZIP31*, which does not contain motif 8. Additionally, *CmbZIP26*, *CmbZIP32,* and *CmbZIP33* had no intron, and they shared the same expression patterns in the process of conidial development, abiotic stress responses, and mycoparasitism, especially *CmbZIP32* and *CmbZIP33*. In contrast, the expression of *CmbZIP31* was not detected by RT-qPCR in the process of conidial development and abiotic stress responses. It could be inferred that *CmbZIP31* may be a pseudogene, resulted from the inserted intron or lack of motif 8.

The conventional bZIP domain harbors a highly conserved basic region with an invariant N-X_7_-R/K motif and a variable leucine-zipper region or several heptad repeats of other bulky hydrophobic amino acids [33]. The basic region is responsible for sequence-specific DNA-binding to regulate gene expression [12]. In this study, we found that the core asparagine (N) residue in 8 out of 34 (23.5%) CmbZIP basic region was replaced by some other amino acids, which is also common in *P. sojae* [59] as well. These mutations would confer novel DNA-binding specificities or characteristics of interaction between the *bZIP* genes and other molecules. In addition to the mutation in the core site of the basic region, four genes (*CmbZIP30*, *-09*, *-25,* and *-34*) showed incomplete bZIP conserved domains, which are also found in *U. virens bZIP* genes [57]. There must be functional redundancy in the *CmbZIP* genes because of the large family members. Therefore, some of the family members might lose some amino acids in the process of evolution. However, due to the functional redundancy of the *bZIP* gene family, some of the *bZIP* genes with the nick did not affect the survival of *C. minitans.* The most conservative site in bZIP domain should be arginine (R) of the invariant N-X7-R motif, only one gene *CmbZIP30* has mutation at this site. 

Previous reports have revealed that introns are involved in gene expression and evolution [67]. Usually, intron/exon arrangement took along the imprint of the evolution, and intron loss is faster than intron gain after segmental duplication [68,69]. In this study, intron analysis showed that 28 *CmbZIP* genes harbored a variable number of introns, and 15 of them shared a feature that introns are inserted in the front of the leucine-zipper region (Figure 4). It is worth noting that most of the intron insertion sites were arginine (R). This indicated that the intron position in bZIP domain was highly conserved, which was related to recognize the arginine (R) and could be treated as a genomic imprinting.

Basic leucine zipper (bZIP) protein located at the center of key pathways regulating cellular decisions [70] and individual bZIP proteins might carry out distinct but overlapping biological functions [71]. In our expression profile data, individual bZIP proteins had a distinct expression pattern in different conditions, indicating they may have diverse functions. In *A. nidulans*, the bZIP transcription factor *FlbB* controls asexual spore formation [48,49]. Most of *CmbZIP* genes were upregulated in the later stages of conidial development (Figure 7), which means some *CmbZIP* genes may be responsible for conidiogenesis. However, mutants of four *CmbZIPs* (*CmbZIP07*, -*09*, -*13,* and -*16*) did not show any significant difference in conidial development. At present, there is no report about the bZIP transcription factor regulating mycoparasitism. In the plant pathogenic fungus *M. oryzae*, three *bZIP* genes (*MobZIP11*, -*13*, and -*22*) were essential for pathogenicity [52]. Our results showed that *CmbZIP09* and *CmbZIP13* highly expressed in the later stages of the process of mycoparasitism (Figure 8). However, the disruption of these two genes had no difference in the parasitic ability. MobZIP21/MoAP1 was identified as a regulator of the oxidative stress response in *M. oryzae* [43,52]. CmbZIP16, an ortholog of MobZIP21/MoAP1, was shown to mediate the oxidative stress response. When *CmbZIP16* was disrupted, disruption mutants showed an increased sensitivity to H_2_O_2_ and VK3. Adapting to low ambient pH, due to oxalic acid secreted by *S. sclerotiorum*, is an important characteristic of *C. minitans* [9,10]. Low pH stress (pH 3) repressed expression of most *CmbZIP*s, but induced expression of *CmbZIP09.* However, the disruption of *CmbZIP09* had no effect on tolerance to oxalic acid. It is possible that other *CmbZIP* genes may compensate the low pH response function in the absence of *CmZIP09*. Likewise, mutants of other two *CmbZIP*s (*CmbZIP07* and *-13*) did not show any significant difference in mycelial growth, conidial production, parasitic ability, and tolerance to the abiotic stress. It is suggested that these *CmbZIP*s might involve in subtle regulation of biological processes or have functional redundancy genes. Therefore, further functional studies of other individual *CmbZIP* genes need to be done for a deeper understanding of this important biocontrol agent.

In conclusion, we systematically identified 34 *bZIP* genes in the *C. minitans* genome, and analyzed the intron arrangement and expression patterns of the *CmbZIP* genes in response to the abiotic stress, during conidial development and mycoparasitism. All data herein suggested that the *CmbZIP* genes play important roles in these processes. Additionally, *CmbZIP16* is required for oxidative stress tolerance. These results provide comprehensive information on the *CmbZIP* gene family and lay the foundation for further functional research of the individual *CmbZIP* genes.

## Supplementary Materials

The following are available online at https://www.mdpi.com/2076-2607/8/7/1045/s1. Figure S1: construction and identification of four *CmbZIP*s knockout mutants: (A) schematic diagram of the *CmbZIP*s deletion strategy and (B) PCR verification of the *CmbZIP*s knockout mutants. *CmbZIP*s knockout mutants were circuited by red roundrect; Figure S2: motif logos of predicted conserved motifs; Table S1: general information about genes encoding CmbZIP transcription factor; Table S2: primers used in this study.
