# Supplementary material for: Genome-Wide Identification and Expression Analysis of the bZIP Transcription Factors in the Mycoparasite Coniothyrium minitans
Source: Microorganisms. 2020 Jul 14;8(7):1045. doi: 10.3390/microorganisms8071045 (PMC7409085; doi:10.3390/microorganisms8071045)
Supplement: Supplementary file 1 [file microorganisms-08-01045-s001.zip › Supplementary files/Table S1. Primers used in this study.docx]

**Table S1. Primers used in this study**

| **Primer name** | **Sequence (5’ → 3’)** |
| --- | --- |
| *CmbZIP1*-F | GGAACCCAAAGGCAACAGTA |
| *CmbZIP1*-R | GGCAAGCCACTGCTTCTTAC |
| *CmbZIP2*-F | CATCATGTTCCTGGTCATGC |
| *CmbZIP2*-R | ATGGAATGAAGAAGGCATCG |
| *CmbZIP3*-F | AATCCAGCTTGAACGACCAC |
| *CmbZIP3*-R | GCCTGCAGTCAAGATGACAA |
| *CmbZIP4*-F | CTGCGTCGAGCCCTATCTAC |
| *CmbZIP4*-R | GGGCTTTGCATTGTACTGGT |
| *CmbZIP5*-F | GACCATGGACAGCTTCGACT |
| *CmbZIP5*-R | GTCGGTGTTCTGGAACTGGT |
| *CmbZIP6*-F | CGACCGACAATTGGTTCTCT |
| *CmbZIP6*-R | CGTGAACCGATGACTTCCTT |
| *CmbZIP7*-F | CCCTACACAACCACCGCTAC |
| *CmbZIP7*-R | GCAATGTCGTCTTCGTGGTA |
| *CmbZIP8*-F | CACTCTGTTGCACCTGTCGT |
| *CmbZIP8*-R | GCCCTTAACCCTTCGATCTC |
| *CmbZIP9*-F | ATGAACGCAGGAAGATCCAG |
| *CmbZIP9*-R | CAGAGAGGTCTCGGTTTTGG |
| *CmbZIP10*-F | ACAGAACCAAACGGATTTGC |
| *CmbZIP10*-R | TCGGGAGCTTGAACTCTTGT |
| *CmbZIP11*-F | ACCAGCGGCTGCTATGTAGT |
| *CmbZIP11*-R | CTCAGCCAACATCTCGTTCA |
| *CmbZIP12*-F | CGTACGATGCCTTTTCCTTC |
| *CmbZIP12*-R | AGGCTCGTCTCCTTCTCCTC |
| *CmbZIP13*-F | CCACCGGATCTGTCAAAAGT |
| *CmbZIP13*-R | GTCGTGATCGGAACCAAACT |
| *CmbZIP14*-F | CGGCATCGTTATTCCATCTT |
| *CmbZIP14*-R | TACCTCTCTTCCGTGGCTGT |
| *CmbZIP15*-F | CAACAATAACGGTGGCCTCT |
| *CmbZIP15*-R | GACTGCAATTGCTGACCTGA |
| *CmbZIP16*-F | TCAGCTCCATGCTGAACAAC |
| *CmbZIP16*-R | GTCCTGTGCTGTCTTGACGA |
| *CmbZIP17*-F | AGGGCTACAGCTCCCTCTTC |
| *CmbZIP17*-R | CGCAGCATTGTAAAGGTCAA |
| *CmbZIP18*-F | CTTGCACCCACAATACAACG |
| *CmbZIP18*-R | TGCTCCATCATCTGCTGTTC |
| *CmbZIP19*-F | ACCAAAGACCACATCGAAGG |
| *CmbZIP19*-R | TGTCCAGCATGAGAAAGACG |
| *CmbZIP20*-F | AAATCAACCATCCTCGAACG |
| *CmbZIP20*-R | CGTTTGACTGGAAGGGATGT |
| *CmbZIP21*-F | GAAAAGCGAAAAGCCAACAC |
| *CmbZIP21*-R | CTCGACCTCCCCCTCAAG |
| *CmbZIP22*-F | GCAGTTCCAACAGCCTCTTC |
| *CmbZIP22*-R | CAACGCTGGCTGTGAAGTTA |
| *CmbZIP23*-F | AAGAAGAAGCACACCGAGGA |
| *CmbZIP23*-R | GCTCTTCCTTGTCGAAGTGG |
| *CmbZIP24*-F | AAGTCACGCCACCAGAAGAC |
| *CmbZIP24*-R | GGTTCTTGGTCGAAGACTGC |
| *CmbZIP25*-F | ACCAACCTCTCCGTCCTGTA |
| *CmbZIP25*-R | CTCTCTTCCCGCAATTCATC |
| *CmbZIP26*-F | ATGAAACTCGCCAGAACACC |
| *CmbZIP26*-R | TTGGTAGCGGTAAAGGATGG |
| *CmbZIP27*-F | CTTGTACACGCCACCACAAC |
| *CmbZIP27*-R | GCATTCGAGATTCCCATGTT |
| *CmbZIP28*-F | TCCGCATCAAGGAATCTTTC |
| *CmbZIP28*-R | TGACACGCTACCAGATGAGC |
| *CmbZIP29*-F | CAACCTCAACACCATCATGC |
| *CmbZIP29*-R | TCTATTGCGTCCATGCTGTC |
| *CmbZIP30*-F | GGTTGACAAGGACACCGTCT |
| *CmbZIP30*-R | CGACTGGGATGACAGTGCTA |
| *CmbZIP31*-F | CCGTACTGTACCCCGACCTA |
| *CmbZIP31*-R | CGGACAGGCCAAGATCTCTA |
| *CmbZIP32*-F | CCATGTCAACTTTGCACCAC |
| *CmbZIP32*-R | TCCTGTTGCCTCTTCTGCTT |
| *CmbZIP33*-F | AGCCGATAAGTGCAGGAGAA |
| *CmbZIP33*-R | TCGCCTCGAGTAGACAACCT |
| *CmbZIP34*-F | AGAGTGGATTCGCAGCTTGT |
| *CmbZIP34*-R | TTGCTCTCATTCGATTCACG |
| *CmActin*-F | TCGTGACTTGACCGACTACCTC |
| *CmActin*-R | TTGCCAATGGTGATGACCTGA |
| P1-bZIP7 | GCCAACAACCAGAAACCACC |
| P2-bZIP7 | CTCCACTAGCTCCAGCCAAGGGAAGAAGAGAAGGAGGAAGC |
| P3-bZIP7 | GCTTCCTCCTTCTCTTCTTCCCTTGGCTGGAGCTAGTGGAG |
| P6-bZIP7 | CGAATGCGCGAAAAGTCGCTCTATTCCTTTGCCCTCGGAC |
| P7-bZIP7 | GTCCGAGGGCAAAGGAATAGAGCGACTTTTCGCGCATTCG |
| P8-bZIP7 | CGGATGCGTCCTCGTAAATC |
| Veri-bZIP7-5’-F | ACCGTGTGCAGTCTTGTCTTC |
| Veri-bZIP7-3’-R | GCAGGTGAAGGTGCCCATAC |
| P1-bZIP9 | ACGCGTCCTAATTTCGACCATG |
| P2-bZIP9 | CTCCACTAGCTCCAGCCAAGGGCTACTTGTTGGTTGATGCTG |
| P3-bZIP9 | CAGCATCAACCAACAAGTAGCCCTTGGCTGGAGCTAGTGGAG |
| P6-bZIP9 | GGCTGATTCGGAACAAAGACAACCTATTCCTTTGCCCTCGGAC |
| P7-bZIP9 | GTCCGAGGGCAAAGGAATAGGTTGTCTTTGTTCCGAATCAGCC |
| P8-bZIP9 | GTCGTTGGGCGTTGTTCATG |
| Veri-bZIP9-5’-F | CTTTGTGCGTATGCTGGCTC |
| Veri-bZIP9-3’-R | CACTGCCAATGCCATGTTACAG |
| P1-bZIP13 | GAGCATACCGCCTCTGTTATTG |
| P2-bZIP13 | CTCCACTAGCTCCAGCCAAGGACGGTGATAAAGACTTCGCC |
| P3-bZIP13 | GGCGAAGTCTTTATCACCGTCCTTGGCTGGAGCTAGTGGAG |
| P6-bZIP13 | GGCGAAGTCTTTATCACCGTCCTTGGCTGGAGCTAGTGGAG |
| P7-bZIP13 | GTCCGAGGGCAAAGGAATAGCTGTAGGCGTTAAGGAGTGTC |
| P8-bZIP13 | GCTACTTGCTACTGTAGGTGG |
| Veri-bZIP13-5’-F | CGTAAACCGATCAAGCACCAC |
| Veri-bZIP13-3’-R | ACCGAGATGGTCAACCCTAG |
| P1-bZIP16 | AGAGTCACTGCGACGTTCC |
| P2-bZIP16 | CTCCACTAGCTCCAGCCAAGCAAAGAACGATCTGGCGGTTG |
| P3-bZIP16 | CAACCGCCACATCGTTCTTTGCTTGGCTGGAGCTAGTGGAG |
| P6-bZIP16 | CCGTGGCCGCTATCTATGTACTATTCCTTTGCCCTCGGAC |
| P7-bZIP16 | GTCCGAGGGCAAAGGAATAGTACATAGATAGCGGCCACGG |
| P8-bZIP16 | CACACACAACCCAAGCACAC |
| Veri-bZIP16-5’-F | AACGATTCTTGCGGGTGCTC |
| Veri-bZIP16-3’-R | CCAATTACACCATTCCCGAGAC |
| P4：(HY5’-R, HY split) | GGATGCCTCCGCTCGAAGTA |
| P5：(HY3’-F, YG split) | CGTTGCAAGACCTGCCTGAA |
| Veri-5’-R | AAGAGTCACACTTCGAGCGC |
| Veri-3’-F | GCAATTTCGATGATGCAGCTTGG |
| HYG-F | ATGAAAAAGCCTGAACTCACCGC |
| HYG-R | CTATTCCTTTGCCCTCGGAC |
