# Supplementary material for: Genome-Wide Identification and Expression Analysis of the bZIP Transcription Factors in the Mycoparasite Coniothyrium minitans
Source: Microorganisms. 2020 Jul 14;8(7):1045. doi: 10.3390/microorganisms8071045 (PMC7409085; doi:10.3390/microorganisms8071045)
Supplement: Supplementary file 1 [file microorganisms-08-01045-s001.zip › Supplementary files/Table S2. General information about genes encoding CmbZIP transcription factor.docx]

**Table S2. General information about *CmbZIP* transcription factor genes**

| Name | cDNA | gDNA | Aa | Exon | MW (kDa) | pI |
| --- | --- | --- | --- | --- | --- | --- |
| *CmbZIP01* | 1644 | 2625 | 547 | 6 | 58.4 | 7.75 |
| *CmbZIP02* | 1548 | 1723 | 515 | 3 | 57.1 | 6.41 |
| *CmbZIP03* | 1077 | 1173 | 358 | 3 | 41.3 | 6.18 |
| *CmbZIP04* | 4743 | 5655 | 1580 | 7 | 175.3 | 6.30 |
| *CmbZIP05* | 912 | 980 | 303 | 2 | 33.3 | 4.85 |
| *CmbZIP06* | 783 | 783 | 260 | 1 | 28.3 | 9.52 |
| *CmbZIP07* | 837 | 994 | 278 | 3 | 31.0 | 6.55 |
| *CmbZIP08* | 1530 | 1707 | 509 | 4 | 56.9 | 6.63 |
| *CmbZIP09* | 420 | 470 | 139 | 2 | 15.7 | 9.80 |
| *CmbZIP10* | 1959 | 2262 | 652 | 3 | 72.4 | 5.81 |
| *CmbZIP11* | 1695 | 2100 | 564 | 4 | 62.0 | 8.14 |
| *CmbZIP12* | 897 | 1046 | 298 | 3 | 32.4 | 5.94 |
| *CmbZIP13* | 696 | 793 | 231 | 3 | 26.2 | 5.36 |
| *CmbZIP14* | 1128 | 1482 | 375 | 5 | 41.8 | 6.93 |
| *CmbZIP15* | 855 | 1049 | 284 | 4 | 31.1 | 6.71 |
| *CmbZIP16* | 1848 | 1946 | 615 | 3 | 66.0 | 4.98 |
| *CmbZIP17* | 1770 | 1821 | 589 | 2 | 65.2 | 4.90 |
| *CmbZIP18* | 699 | 1069 | 232 | 2 | 26.1 | 5.46 |
| *CmbZIP19* | 1176 | 1379 | 391 | 2 | 43.1 | 9.05 |
| *CmbZIP20* | 1263 | 1383 | 420 | 3 | 46.0 | 6.23 |
| *CmbZIP21* | 372 | 419 | 123 | 2 | 13.5 | 10.00 |
| *CmbZIP22* | 1311 | 3518 | 436 | 2 | 46.7 | 8.54 |
| *CmbZIP23* | 1950 | 2045 | 649 | 2 | 72.2 | 5.34 |
| *CmbZIP24* | 978 | 978 | 325 | 1 | 36.0 | 6.5 |
| *CmbZIP25* | 771 | 771 | 256 | 1 | 28.3 | 6.01 |
| *CmbZIP26* | 918 | 918 | 305 | 1 | 33.7 | 6.62 |
| *CmbZIP27* | 831 | 881 | 276 | 2 | 31.2 | 9.36 |
| *CmbZIP28* | 1245 | 1554 | 414 | 5 | 44.9 | 6.09 |
| *CmbZIP29* | 834 | 1239 | 277 | 2 | 30.3 | 5.01 |
| *CmbZIP30* | 1692 | 1897 | 563 | 3 | 60.3 | 5.02 |
| *CmbZIP31* | 762 | 852 | 253 | 2 | 28.7 | 8.82 |
| *CmbZIP32* | 948 | 948 | 315 | 1 | 35.3 | 6.2 |
| *CmbZIP33* | 867 | 867 | 288 | 1 | 32.3 | 6.22 |
| *CmbZIP34* | 825 | 883 | 274 | 2 | 30.6 | 5.59 |
