# Supplementary figures and images for: Genome-Wide Identification and Expression Analysis of the bZIP Transcription Factors in the Mycoparasite Coniothyrium minitans
Source: Microorganisms. 2020 Jul 14;8(7):1045. doi: 10.3390/microorganisms8071045 (PMC7409085; doi:10.3390/microorganisms8071045)

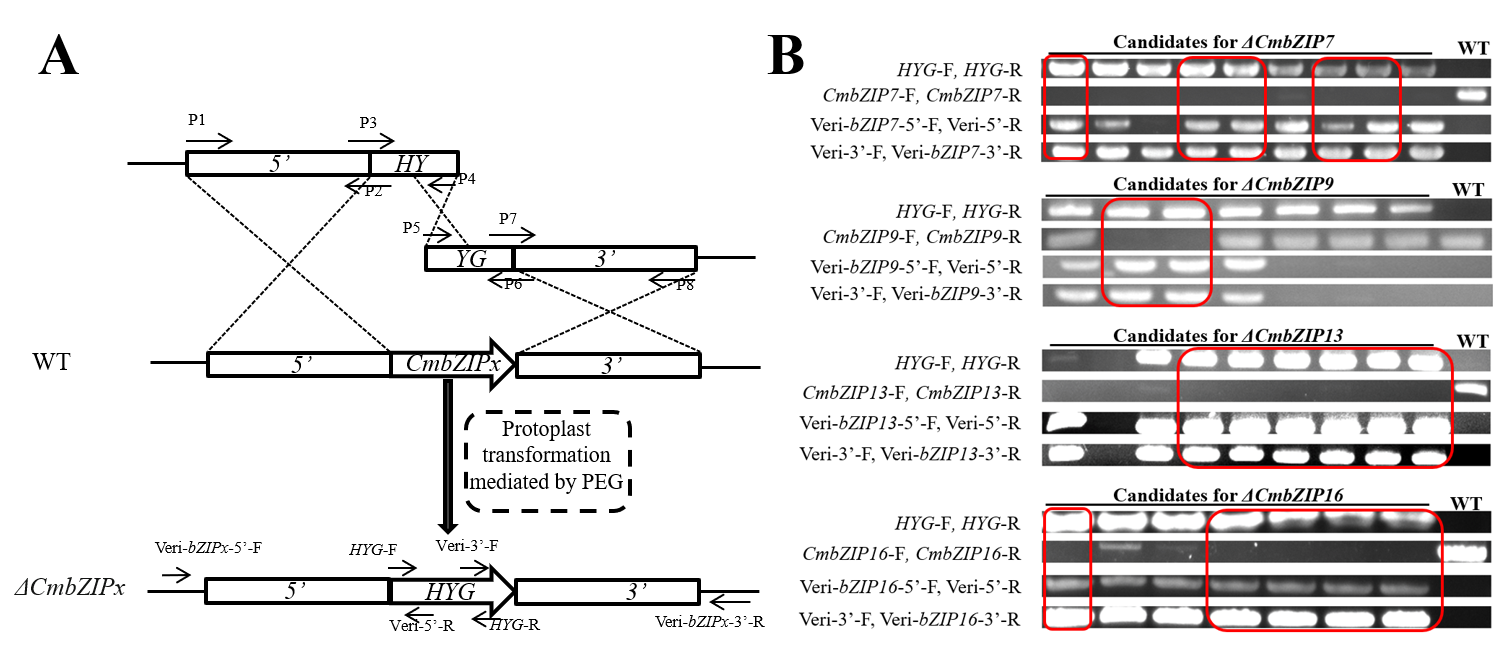

Supplement: Supplementary file 1 [file microorganisms-08-01045-s001.zip › Supplementary files/Figure S1. Construction and identification of individual CmbZIPs knockout mutants.tif]

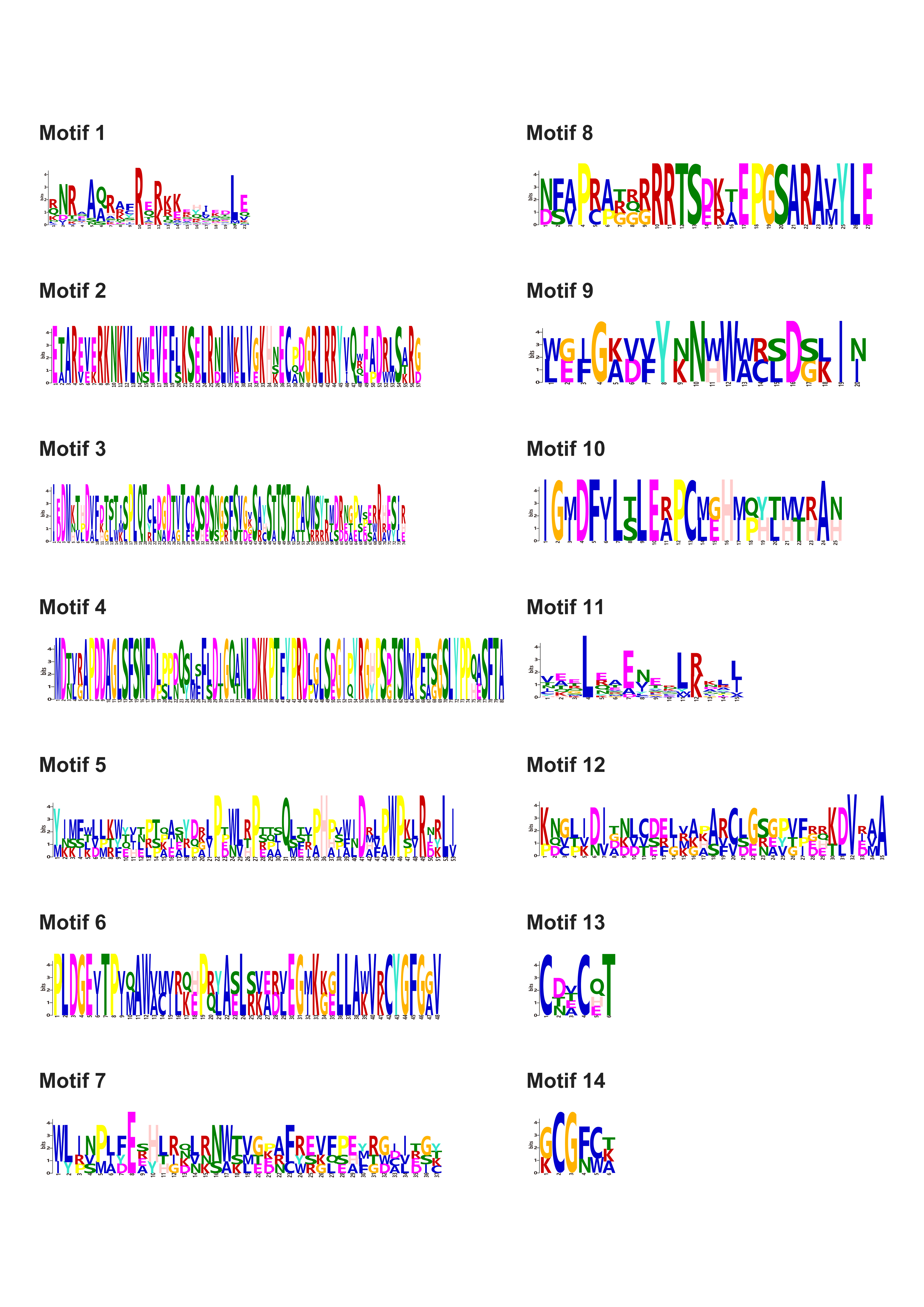

Supplement: Supplementary file 1 [file microorganisms-08-01045-s001.zip › Supplementary files/Figure S2. Motif logos of predicted conserved motifs.jpg]
